# EntoSieve: Automated Size‐Sorting of Insect Bulk Samples to Aid Accurate Megabarcoding and Metabarcoding

**DOI:** 10.1111/1755-0998.14097

**Published:** 2025-03-11

**Authors:** Aleida Ascenzi, Lorenz Wührl, Vivian Feng, Nathalie Klug, Christian Pylatiuk, Pierfilippo Cerretti, Rudolf Meier

**Affiliations:** ^1^ Department of Biology and Biotechnologies “Charles Darwin” Sapienza University of Rome Rome Italy; ^2^ Museum of Zoology (MZUR), Sapienza University of Rome Rome Italy; ^3^ Institute for Automation and Applied Informatics (IAI) Karlsruhe Institute of Technology (KIT) Karlsruhe Germany; ^4^ Museum für Naturkunde, Center for Integrative Biodiversity Discovery, Leibniz‐Institut für Evolutions‐Und Biodiversitätsforschung Berlin Germany; ^5^ Institut für Biologie Humboldt University Berlin Berlin Germany

**Keywords:** biomass bias, bulk samples, DNA barcoding, insect biodiversity, sequence recovery

## Abstract

Widespread insect decline necessitates the development and use of standardized protocols for regular monitoring. These methods have to be rapid, efficient and cost‐effective to allow for large‐scale implementation. Many insect sampling and molecular methods have been developed. These include Malaise trapping, high‐throughput DNA barcoding (‘megabarcoding’) and metabarcoding. The latter allows for assessing the species diversity in whole samples using few steps, but sample heterogeneity in terms of body size remains a challenge since large insects contribute disproportionately more mtDNA than small ones. This can potentially overwhelm the template DNA from small species that then go undetected. Size‐sorting can mitigate this problem, but no satisfying automated, rapid and non‐destructive solutions are available. We introduce the EntoSieve, a low‐cost and DIY motorized instrument that disentangles and sorts abundant insect bulk samples into several body size fractions while minimizing damage to specimens, thus reducing the risk of DNA contamination across size fractions (e.g. legs of large specimens in small body size fraction). EntoSieve utilizes readily available components, 3D‐printed parts and customizable meshes, thus enabling parallelization at low cost. We here show the efficiency of the EntoSieve for three samples with more than 10,000 specimens using three sieving protocols and assess the impact on specimen integrity. Efficiency ranged from 92% to 99%, achieved within 18–60 min, and specimen damage was not significant for subsamples. By facilitating rapid pre‐processing, the device contributes to producing morphologically valuable vouchers for megabarcoding and is likely to improve compositional diversity accuracy across size classes when using metabarcoding.

## 1 Introduction

Insects are the most species‐rich taxon of Metazoa (Chapman 2009) and play crucial roles in ecosystems, thus making them an essential element of biodiversity. However, most insect species are difficult to monitor in part because most are still undescribed (Stork 2018). Given the importance of insect communities for ecosystem health, insects have recently nevertheless become the focus of many research projects studying insect decline (Hallmann et al. 2017; Lister and Garcia 2018), and standardized protocols for collecting have been developed to identify the drivers of insect decline (Mora et al. 2011; Outhwaite et al. 2020; Meier et al. 2024).

However, most standardized collecting methods (e.g. Malaise traps) yield thousands of specimens belonging to hundreds of species within 1–2 weeks of collecting (Perez et al. 2015; Srivathsan et al. 2019; Karlsson et al. 2020). The specimens furthermore represent a large range of body sizes (Chown and Gaston 2010; Chimeno et al. 2023), so the processing of standardized samples is not trivial. Traditionally, the specimens were sorted to morphospecies level. Subsequently, only a few specimens of each morphospecies were selected for barcoding (Ranasinghe et al. 2022; see also Riedel et al. 2013). However, this approach is expensive when applied to entire samples and leads to unpredictable errors related to morphospecies sorting (Oliver and Beattie 1993; Krell 2004; Yu et al. 2012). ‘Cherry‐picking’ only a few selected specimens furthermore left most specimens unprocessed, thus overlooking many cryptic species (Hebert et al. 2004; Jörger and Schrödl 2013; Williams et al. 2022). This can have cascading effects on the quality of biodiversity assessments (Bortolus 2008). This is a particularly serious problem when the so‐called ‘dark taxa’ (Page 2016; Hausmann et al. 2020; see Hartop et al. 2022 for a formal definition) are ignored, since they dominate Malaise trap samples worldwide (Hartop et al. 2022; Srivathsan et al. 2023). These groups consist of highly diverse taxa, primarily composed of small‐bodied organisms with incompletely documented species‐level diversity (e.g. Hausmann et al. 2020). Note that using traditional taxonomic methods for species identification is particularly challenging for such dark taxa (Morinière et al. 2019).

Fortunately, the proposal of DNA barcoding (Hebert et al. 2003) combined with the advent of high‐throughput sequencing (HTS) has significantly enhanced our ability to analyse bulk invertebrate samples by reversing the traditional workflow (Wang et al. 2018). Instead of sorting the specimens to morphospecies using morphology and then validating the morphospecies by barcoding a few specimens for each, one can now reverse the process by barcoding all specimens and then validating Molecular Taxonomic Units (MOTUs) through morphological inspection based on a few specimens for each MOTU (see Wang et al. 2018; Yeo et al. 2020; Hartop et al. 2022; Caruso et al. 2024; Vasilita et al. 2024). Furthermore, image‐based sorting techniques are on the horizon that will process many specimens in bulk samples by assigning individual specimens to taxa through the analysis with convolutional neural networks (CNNs) applied to images (Feng et al. 2016; Perre et al. 2016; Ärje et al. 2020). Similarly, robots like the DiversityScanner (Wührl et al. 2022) expedite the sorting of individual specimens (< 3 mm) prior to molecular analysis by sorting them into different classes based on images and transferring them into a 96‐well microplate, thus preparing them for sequencing.

A crucial initial step in processing bulk samples is the size sorting and disentangling of specimens, which divides bulk samples into multiple consistent size fractions that can then be handled by robots designed for specimens of certain body sizes. Homogeneous samples, in terms of body size, facilitate molecular analysis and automated identification, but also traditional taxonomy, because for many taxa, target specimens will be found in only one size class (Haas‐Renninger et al. 2023). Unfortunately, manual size sorting is exceedingly time‐consuming (Danks 1996), and the required time increases with sample heterogeneity. Note that size sorting is also important for metabarcoding because a significant challenge in metabarcoding stems from the varying amounts of DNA obtained from specimens of different sizes (Deagle et al. 2018; Elbrecht et al. 2021). Larger insects tend to contain a higher number of mitochondrial genome copies (Burgess et al. 2017; Bista et al. 2018) and provide more concentrated DNA templates, dominating DNA extracts of bulk samples, while small and soft‐bodied specimens tend to be underrepresented in bulk when the DNA of large and highly sclerotized taxa is simultaneously extracted. This renders it necessary to increase DNA template volumes per specimen for soft‐bodied and/or small specimens (Zizka et al. 2022), especially when these taxa are not abundant (Strutzenberger et al. 2024). Disproportionate DNA apportions can then lead to false negatives (low DNA‐yield input taxa), altering species presence/absence assessments. This impedes the interpretation of metabarcoding data, which are already affected negatively by primer biases, leading to some taxa being more readily amplified than others (Elbrecht and Leese 2015; Arribas et al. 2016; Krehenwinkel et al. 2017). One method that has been proposed to alleviate these undesirable effects is the processing of size fractions consisting of specimens of similar size, which is thought to reduce the likelihood of missing small‐bodied species (Elbrecht et al. 2017). Furthermore, this approach presumably also reduces the sequencing depth required to detect rare and small‐bodied species (Zhou et al. 2013; Braukmann et al. 2019), ultimately reducing sequencing costs (Elbrecht et al. 2017). Size sorting has thus been recommended as a default initial step by many metabarcoding protocols (Table 1) for addressing issues related to body size heterogeneity and can be further improved when it is combined with proportional pooling based on taxon body size distribution and/or taxon abundance (Morinière et al. 2016; Creedy et al. 2019; Buchner, Haase, and Leese 2021; Elbrecht et al. 2021).

**TABLE 1:** A selection of studies that size‐sorted bulk or mock invertebrate samples for metabarcoding.

Reference	Methodology	Test	Taxon	Sample composition	Sampling method
Buchner, Beermann, et al. (2021)	Metabarcoding	PCR volumes efficiency	Arthropods	Mock vs. bulk	Malaise trap
Buchner, Haase, and Leese (2021)	Metabarcoding; metagenomics	Sample homogenization	Invertebrates	Bulk	Malaise trap and kick‐net
Creedy et al. (2019)	Metabarcoding	Biodiversity assessment	Arthropods	Bulk	Canopy fogging
Decker et al. (2024)	Metabarcoding	Biodiversity assessment	Arthropods	Bulk	Malaise trap
Elbrecht and Leese (2015)	Metabarcoding	Primer bias and biomass‐sequence relationships	Freshwater macroinvertebrates	Mock	Hand sampling
Elbrecht et al. (2017)	Metabarcoding	Effect of specimen biomass	Freshwater macroinvertebrates	Mock	Kick‐net
Elbrecht et al. (2021)	Metabarcoding	Body size proportional amplicon pooling	Arthropods	Bulk	Malaise trap
Huang et al. (2022)	Metabarcoding	Biodiversity assessment	Dipterans	Bulk	Malaise
Iwaszkiewicz‐Eggebrecht et al. (2023)	Metabarcoding	DNA extraction protocols	Insects	Mock	Cultures
Kerner et al. (2024)	Metabarcoding	Biodiversity assessment	Insects	Bulk	Malaise trap vs. hand netting
Kirse et al. (2023)	Metabarcoding	Destructive and non‐destructive DNA extraction methods	Arthropods	Bulk	Malaise trap
Kortmann et al. (2021)	Metabarcoding	Biodiversity assessment	Insects	Bulk	Malaise trap
Kortmann et al. (2022)	Metabarcoding	Biodiversity assessment	Insects	Bulk	Malaise trap
Köthe et al. (2023)	Metabarcoding	Biodiversity assessment: insect conservation	Insects	Bulk	Malaise trap
Krehenwinkel et al. (2018)	Metabarcoding	DNA degradation bias	Arthropods and microbiota	Mock	Beating sheet
Leray and Knowlton (2015)	Metabarcoding	Biodiversity assessment	Marine benthic invertebrates	Bulk	Artificial Reef Monitoring Structures (ARMS)
Lim et al. (2022)	Metabarcoding	Biodiversity assessment	Arthropods	Bulk	Beating sheet
Remmel et al. (2024)	Metabarcoding	Biodiversity assessment	Insects	Bulk	Malaise
Röder et al. (2024)	Metabarcoding	Control of vector species: effect of Bti on body size distribution	Chironomidae (Insecta, Diptera)	Bulk	Floating emergent trap
Yang et al. (2021)	Metabarcoding	Overall pipeline	Arthropods	Mock	Malaise trap
Zhou et al. (2013)	Metabarcoding	Ultra‐deep sequencing without PCR	Insects	Bulk	Light trap
Zizka et al. (2022)	Metabarcoding	Subsampling effect	Arthropods	Bulk	Malaise trap

To our knowledge, there are currently only two standardized methods for automated size sorting (Buffington and Gates 2008; Elbrecht et al. 2021), both based on size fractioning through sieving. The first is the Fractionator (Buffington and Gates 2008). In this method, a shaker is used to sieve a bulk sample through a 2 mm mesh strainer placed inside a box with water and a few drops of dish soap to reduce surface tension and facilitate specimens disentangling. This approach produces two size fractions with a success rate of 57%–100% on small Hymenoptera in 30 min (Haas‐Renninger et al. 2023). After sieving, the large fraction is collected using tweezers or by spraying the strainer with water. The second automated sieving method stacks sieve containers with multiple mesh sizes on a shaker (Elbrecht et al. 2021). Size sorting can either be carried out under dry or wet conditions in ethanol. The wet method was better at size sorting but less effective in disentangling specimens (Elbrecht et al. 2021). It was also costly and potentially hazardous because it required large amounts of ethanol. In contrast, dry size sorting had lower accuracy, caused more damage to the specimens and took longer due to the electrostatic charging of specimens (2–3 h).

However, preventing damage to specimens during size sorting is important for several applications. In homogenization‐based metabarcoding, the presence of broken body parts in a sample fraction can potentially bias diversity assessments (Leray and Knowlton 2015; Wangensteen and Turon 2016). Specimen damage is even more unwanted for metabarcoding of DNA obtained with mild‐lysis protocols and megabarcoding, where vouchers are preserved for follow‐up research. For example, high‐quality barcodes can be obtained through NGS techniques (Meier et al. 2016; Srivathsan et al. 2021) to achieve species‐level validated identifications. This is feasible if preliminary species hypotheses based on barcodes are validated by protocols such as the one described in Hartop et al. (2022). Intact vouchers are furthermore of critical importance for obtaining high‐quality images for subsequent morphological study, taxonomic revisions (Shim and Song 2024), morphometrics (Xi et al. 2024), training of AI models (Shirali et al. 2024) and downstream research using DNA for determining genetic diversity (Foote et al. 2016), assembling genomes and reconstructing phylogenetic relationships (Magnussen et al. 2022).

Here, we introduce the ‘EntoSieve’, a motorized device designed to sort bulk samples into multiple fractions based on body size. This semi‐automated device is constructed using readily available elements and 3D‐printed parts. The EntoSieve operates with water to minimize fire risk and reduce overall processing costs. It efficiently, rapidly and gently size‐sorts large and heterogeneous bulk samples (i.e. ~10,000 specimens). We tested the device on three abundant and diverse Malaise trap samples and present results on the EntoSieve's efficiency in sieving and the preservation status of the sorted specimens, with a focus on size and taxonomic order.

## 2 Materials and Methods

### 2.1 Robot Concept

**FIGURE 1:**
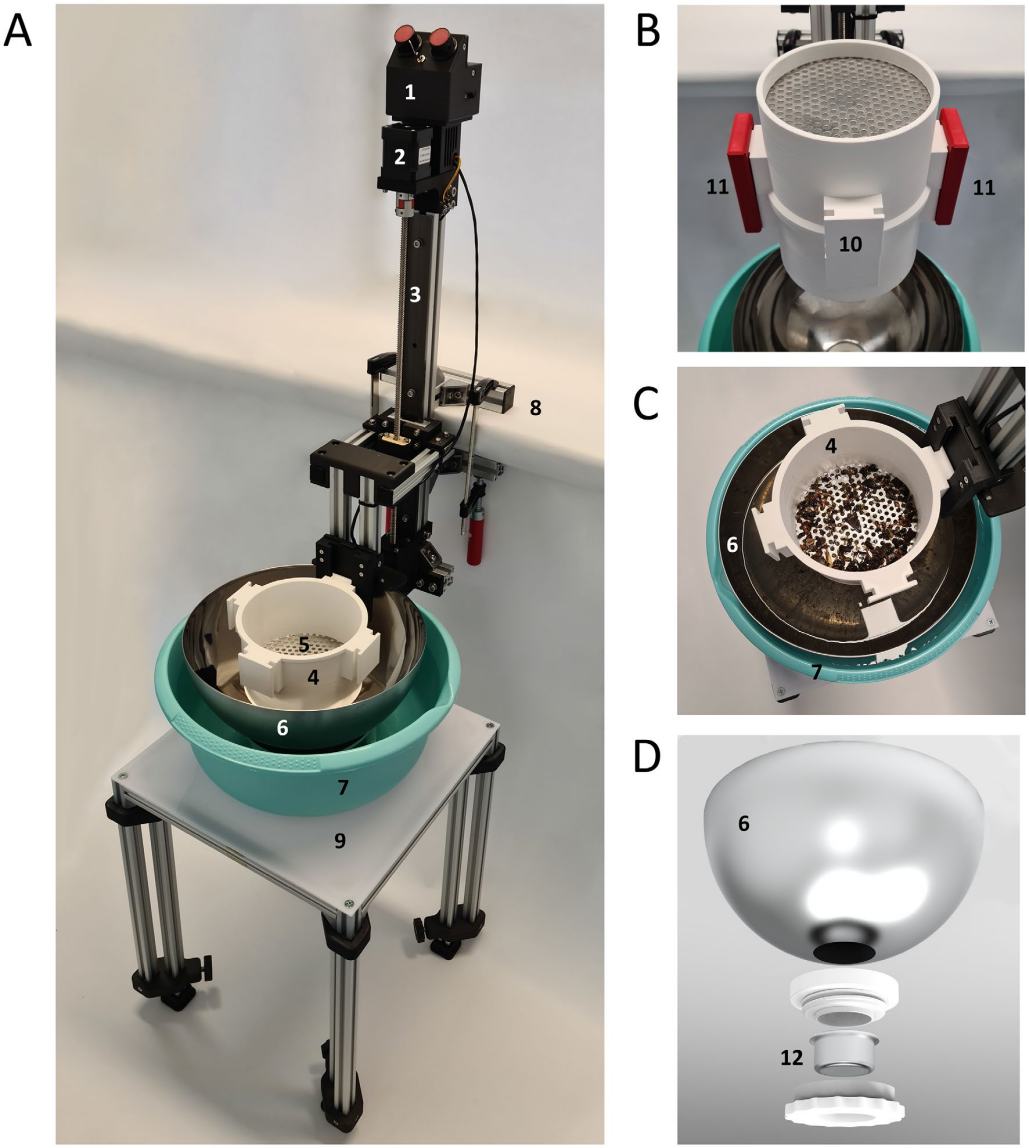
(A) EntoSieve with (1) microcontroller and rotary controls for setting speed and stroke, (2) stepper motor, (3) linear rail with spindle drive, (4) sieve container, (5) perforated sieve, (6) inner container, (7) outer container. The system is clamped (8) on the edge of a table or bench to ensure stability. The inner and outer containers stand on a (9) height‐adjustable table. (B) Two sieve containers: To quickly transfer the insects for the next size category to a second sieve container by 180° rotation on their horizontal axis, the (10) sieve containers are stackable and can be temporarily fixed in place using (11) clamps. (C) Specimens in the sieve container during sieving. (D) Inner container concept with the (12) coffee filter to separate the insects from the water.

The EntoSieve, shown in Figure 1, is controlled by the programmable microcontroller Arduino Nano that is housed in an electronic box (1). The speed and stroke of the system can be adjusted by two rotary knobs. Upon starting the program, a stepper motor (2) drives a sliding carriage (drylin W carriage WWQ, Igus GmbH, Cologne, Germany) along a hard‐anodized aluminium rail (drylin W double rail WSQ, Igus GmbH, Cologne, Germany) using a lead screw (Ds 8 mm × 10 mm, dryspin, Igus GmbH, Cologne, Germany) with a matching flange nut (dryspin JFRM, Igus GmbH, Cologne, Germany) (3). Initially, the lowest point is determined by a lower reference switch and the highest point by an upper reference switch. Depending on the set parameters, the system starts a continuous up‐and‐down movement of the 3D‐printed sieve container (4).

Widely available and cheap aluminium perforated plates of 1 mm thickness and with different perforation diameters are used as sieves (< 1.00 € per sieve). These plates are cut into circles with a diameter of 138 mm and placed in the sieve container (5). During sieving, insects too large for the perforations remain in the sieve container, while smaller insects are washed into the inner container (6) (see Figure 1D). The inner container has a coffee filter at the bottom, allowing the water to drain into the outer container (7) when the inner container is removed after sieving. The small fraction remains in the filter of the inner container.

The EntoSieve is constructed with aluminium strut profiles (30 mm × 30 mm and 20 mm × 20 mm, Bosch Rexroth AG, Lohr am Main, Germany) and can be clamped to a table with screw clamps (8) for maximum stability. A video of the machine setting and functioning is available at: https://youtu.be/n2pqZGBjDQA. During sieving, the containers are placed on a height‐adjustable table (9), making the system adaptable to different mounting heights. Once sieving with one sieve size is complete, the insects are removed from the inner container, which is then placed back into the outer container. The fraction remaining in the sieve container is then transferred to another sieve container with larger perforations. To do this, a second sieve container is placed on top of the first one filled with insects and secured with clamps, as shown in Figure 1C (11). The sieve containers are then rotated upside down (180° rotation on the horizontal axis), and the insects are washed into the second sieve container, allowing the sieving process to continue with inverted containers.

The electronic setup is kept simple to ensure reproducibility. The system includes an Arduino Nano for control. It features a DC‐DC voltage converter that reduces the input voltage (12–24 V) to the 5 V needed for the Arduino Nano. The stepper motor (Nema 17‐1, SIMAC Electronics GmbH, Neukirchen‐Vluyn, Germany) is connected to the Arduino via an A4988 stepper motor driver (Pololu Corporation, Las Vegas, USA). Two end‐stop switches are used for axis referencing, which sets the lowest point of the movement. Additionally, two potentiometers are read via analogue inputs on the Arduino to control the system's speed and amplitude. An RGB‐LED indicates the system's status. The total cost of all components required to set up an EntoSieve, including materials for 3D printing (all plastic parts; Figure 1: components 1, 2, 3, 4, 10, 11 and 12), is approximately €400, making it an affordable solution.

### 2.2 Machine Functioning

Before sieving a bulk sample, we recommend manually removing any extra‐large specimens (i.e. longer than 30 mm) to prevent obstruction of mesh holes or entangling smaller insects, which could hinder efficient fractioning. The EntoSieve is installed on the working surface, with the inner and outer containers positioned beneath the system. Ensure that the coffee filter of the inner container adheres well to the bottom of the outer container (i.e. that the entire bottom surface of the coffee filter touches the outer container) to prevent leakage during sieving. Thus, both the coffee filter and the outer container should have a flat bottoms. The smaller sieve is used first, followed by sieves of increasing sizes, which minimizes manual steps in recovering fractions. To start the sieving process, lower the sieve container to its lowest position. Then, gently pour water into the containers, adjusting the level 30–50 mm above the perforated sieve—for our setup, we used 3.5–4.0 L of water. To avoid damaging the specimens and ensure that insects are directly immersed without hitting the sieve, pour the bulk sample into the sieve container at this setting. Next, adjust the water level to fully submerge the specimens. The system's speed ranges from 34 to 104 mm/s; we recommend setting it to the maximum speed to guarantee optimal results. Adjust the maximum height of the container through the right rotatory knob on the microcontroller (Figure 1, component 1) according to the water level so that the sieve container fully emerges with each oscillation. This allows water to flush up through the holes as the sieve container descends, stirring the specimens and facilitating their separation.

Sieving time should be adjusted based on sample characteristics. The sieve container is equipped with four handles to allow 45° rotations on the vertical axis, which helps shuffle the sample in case specimens cluster on one side of the sieve container while sieving. Additionally, for more effective sieving of especially abundant samples, gently mix the specimens while they are floating. This step can also be useful if certain taxa, such as butterflies or aphids, create a film on the water surface, preventing smaller specimens from being processed.

To retrieve the sieved specimens, stop the system at its highest point, move the containers away from the system, lift the inner container and place it on a stand. Remove the coffee filter and tap it on absorbent paper to remove excess water. Collect the size‐sorted specimens by inverting the coffee filter into a Petri dish or a tube, and gently remove specimens by spraying them with ethanol. To sieve larger specimens, install the next larger sieve, flip the sieve containers and repeat the process. Finally, the larger fraction left in the sieve is poured into the inner collection container and retrieved as described.

Unwanted insect body fragments may remain attached to the EntoSieve surfaces after use, which could cause cross‐contamination with the next sample. Contamination is a well‐documented concern in metabarcoding workflows (McKnight et al. 2019; Batovska et al. 2021; Bohmann et al. 2022), especially for low‐biomass samples (Salter et al. 2014). Effective decontamination of surfaces (i.e. blenders, filters, etc.) typically involves a combination of mechanical cleaning, chemical treatments and, where applicable, high‐temperature or UV sterilization (Prince and Andrus 1992; Hajibabaei et al. 2019; Buchner, Haase, and Leese 2021; Kirse et al. 2023). Following these protocols, we discuss what materials are used by all EntoSieve components and how they can be decontaminated using protocols recommended for metabarcoding studies.

**FIGURE 2:**
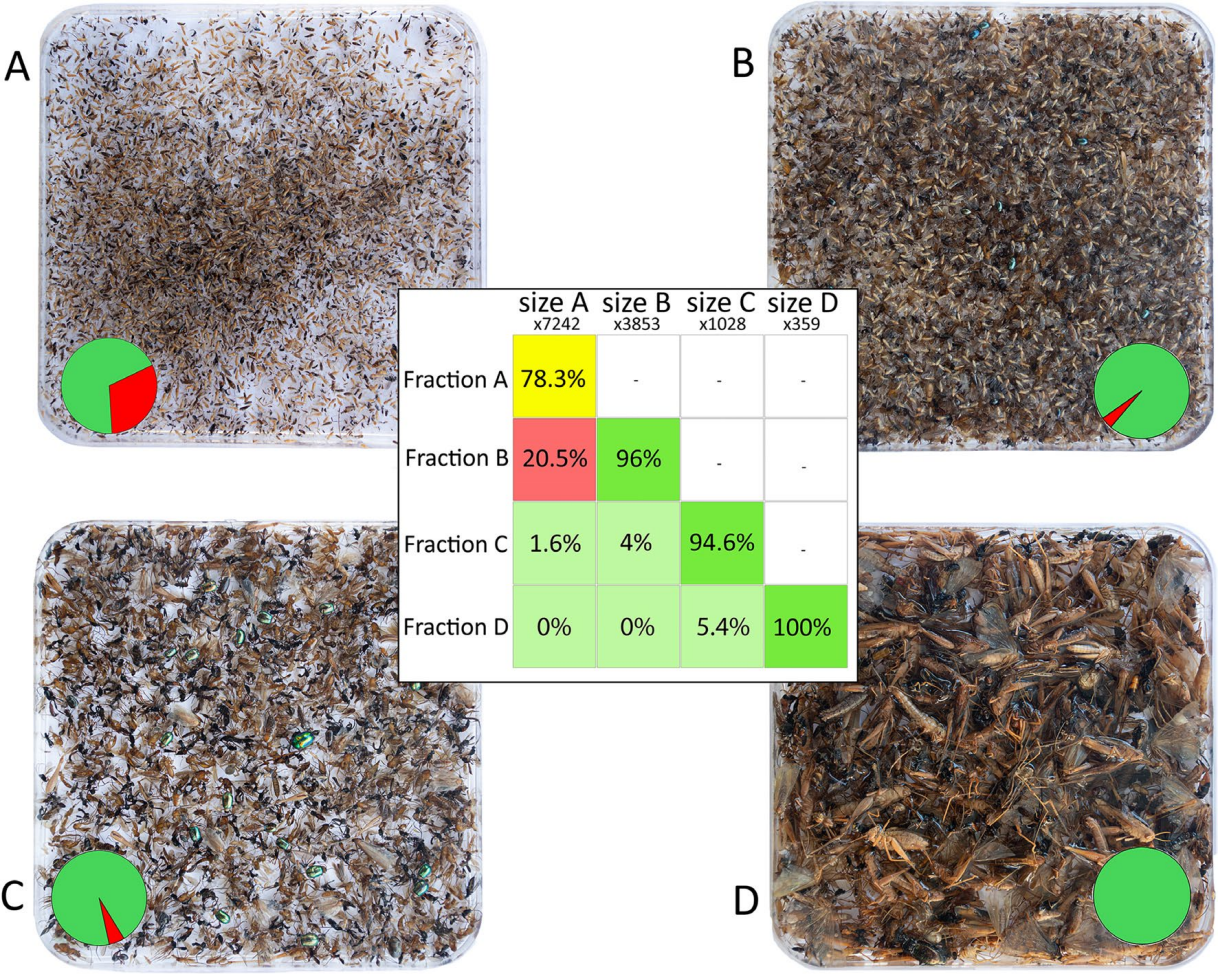
The four size fractions separated in test 2 using meshes MA = 1.5 mm; MB = 3 mm; MC = 5 mm for 20 min each. (A) Fraction A; (B) fraction B; (C) fraction C; (D) fraction D. Pie charts illustrate the ratio of correctly sieved specimens (green) and intruders (red) of each size category. The table shows the percentage of specimens of each size category (columns) that were sorted into each fraction (rows). Numbers on columns' names indicate the total number of specimens.

**FIGURE 3:**
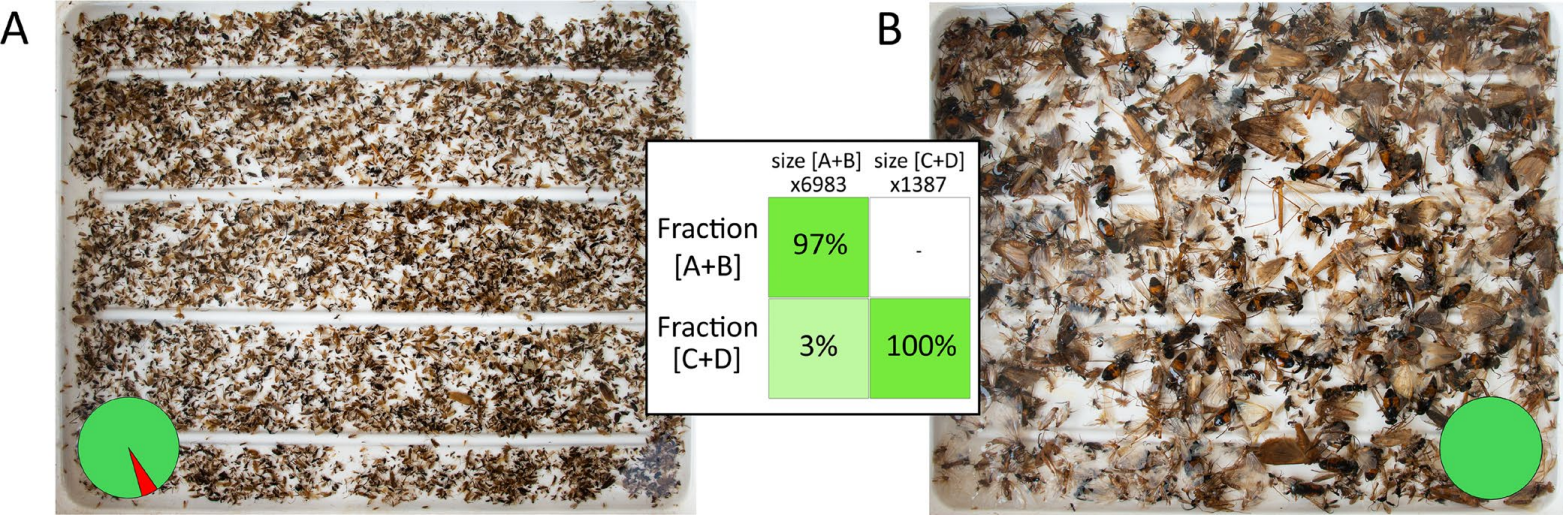
The two size fractions separated in test 3 using sieve MB = 3 mm for 20 min. (A) Fraction [A + B]; (B) fraction [C + D]. Pie charts illustrate the ratio of correctly sieved specimens (green) and intruders (red) of each category. The table shows the percentage of specimens of each size category (columns) that were sorted into each fraction (rows). Numbers on columns' names indicate the total number of specimens.

The following parts of the EntoSieve come into direct contact with biological material: the sieve containers made of polylactic acid (PLA) (Figure 1A, 4), the perforated sieve made of aluminium (Figure 1A, 5), the inner collection container with filter mount made of stainless steel, PLA and silicone (Figure 1A, 6), the outer collection container made of polypropylene (PP) (Figure 1A, 7) and the filter made of stainless steel (Figure 1D, 12). These components should undergo mechanical decontamination through washing followed by chemical decontamination. This can be done either by spraying them with a 5%–10% bleach (NaClO) solution (Fernández et al. 2018) or by immersing them in the solution for 15 min (Majaneva et al. 2018). Alternatively, less corrosive products such as DNA‐Erase, DNA AWAY, DNA/RNA‐ExitusPlus or ELIMINase can replace bleach. A subsequent rinse with distilled water and 70% ethanol is recommended. The aluminium components can be autoclaved (personal observation) or sterilized using UV treatment (Hajibabaei et al. 2019; Erdozain et al. 2019). To minimize downtime during decontamination, it is advisable to have duplicates or triplicates for all critical components (Figure 1: 4, 5, 6, 7, 10, 12). Other components that do not come into direct contact with biological material require less cleaning when gloves are used. These include the microcontroller and rotary knobs (PLA and PP) (Figure 1A, 1), stepper motor (PLA‐coated steel and aluminium) (Figure 1A, 2), linear rail with spline drive (hardened anodized aluminium, stainless steel and iglidur) (Figure 1A, 3), height‐adjustable table (anodized aluminium, PLA, polycarbonate and stainless steel screws) (Figure 1A, 9) and clamps (chromed steel, cast iron and wood handle) (Figure 1B, 11). For these components, mechanical decontamination can consist of compressed air spraying, while chemical decontamination should involve treatment with a 5%–10% NaClO solution, followed by distilled water rinsing and a final treatment with 70% ethanol spray. By following this protocol, DNA contamination can be significantly reduced, ensuring the accuracy of metabarcoding analyses.

### 2.3 Tests

After initially evaluating a system based on shakers and a rotating sieve, we developed a much gentler system using only an up‐and‐down movement. This was tested using three bulk samples (Table 2). We established (I) the system's efficiency by calculating the ratio of correctly sieved specimens in each size category (i.e. insects that ended up in the expected size fraction)—*test 1*, *test 2* and *test 3*—and (II) the damage to specimens (i.e. broken or missing body appendages) by assessing the morphological integrity before and after sieving—*test 1*.

**TABLE 2:** EntoSieve testing. Test purpose and metadata for bulk samples.

Test code	Sample code	Sample information	Habitat features	Variable tested	Mesh size	Sieving time
*Test 1*	*Sample 1*	Castelporziano (Rome, Italy); 4‐21/VI/2022; 41.779497, 12.412719	Pastoral areas for livestock interspersed with wetlands; near sea level; Mediterranean climate	Efficiency, damage	1.5 mm > 3.0 mm > 5.0 mm	6′ > 6′ > 6′
*Test 2*	*Sample 2*	Chiarano‐Sparvera forest (Abruzzo, Italy); 05‐20/VII/2019; 41.858134, 13.963788	Ecotonal zone between forest and grassland; at 1650 m a.s.l.; Apennine temperate mountain climate	Efficiency	1.5 mm > 3.0 mm > 5.0 mm	20′ > 20′ > 20′
*Test 3*	*Sample 3*	Chiarano‐Sparvera forest (Abruzzo, Italy); 05‐20/VII/2019; 41.858052, 13.962609	Ecotonal zone between forest and grassland; at 1650 m a.s.l.; Apennine temperate mountain climate	Efficiency	3.0 mm	20′

The samples were stored at the Museum of Zoology of Sapienza University of Rome (MZUR) in ethanol 96% at room temperature. All three samples were heterogeneous in body size and abundance, containing 10,276, 12,482 and 8370 specimens, respectively. Only very large insects (> 30 mm in length) were manually removed before each test. We employed three sieves with different mesh sizes: ‘MA’ (1.5 mm), ‘MB’ (3 mm) and ‘MC’ (5 mm), used sequentially from smallest to largest. Four body size fractions (referred to as ‘size categories’ or ‘categories’ hereafter) were defined based on insect body lengths: A < 1.5 mm; B = 1.5–3.0 mm; C = 3.0–5.0 mm; D > 5.0 mm.

In *test 1*, we used sieves MA, MB and MC for 6 min each to assess both system efficiency and possible damage to specimens. In *test 2*, we employed the same sieves but increased the sieving time to 20 min per mesh to evaluate any changes in efficiency. In *test 3*, we only used MB to simulate a quicker disentangling process, separating the sample into two fractions: (I) [A + B] < 3.0 mm; (II) [C + D] > 3.0 mm, reflecting a common sieving approach in metabarcoding (Elbrecht et al. 2021).

Additionally, we recorded the time required to remove very large specimens and the time needed for water to drain from the inner to the outer container to determine the total duration of each sieving session. All data are available from https://osf.io/6t25n/.

#### 2.3.1 Efficiency Tests

Efficiency was assessed by estimating the percentage of specimens in each size category that were correctly sieved (i.e. that ended up in the size fraction corresponding to the mesh hole size). Specimens sieved into the wrong size category are referred to as intruders. For each test, we calculated the number of intruders from different size categories in each fraction. For example, in *test 1*, the efficiency for category A was calculated as follows: Efficiency of A = 100 − (number of intruders A × 100/total number of specimens A). We then calculated the overall efficiency of each test by averaging the efficiency across all size fractions. To minimize further handling and potential damage to the specimens, we counted correctly sieved specimens and intruders from overview photographs. Specimens were gently spread inside squared Petri dishes, and photographs were taken from a fixed height of 305 mm by using a Nikon D7100 with an AF‐S DX NIKKOR 18–140 mm f/3.5–5.6 G ED VR lens. The images were processed using a licensed copy of Adobe Photoshop (Adobe Systems Incorporated, San Jose, CA, USA). Insects from different size categories were marked with dots of distinct colours (A: red, B: blue, C: green, D: magenta) using an 18 pixel brush (an example of count is shown in Figure S1). The standardized brush size and the fixed height enabled precise measurement of the specimens for accurate colour assignment. If there was any uncertainty about the size or quantity of specimens in a section of the image, we cross‐verified the Petri dish. The dots of each colour were individually selected and counted using the ‘record measurements’ function in Adobe Photoshop, providing counts of intruders and correctly sieved insects. In *test 3*, only two size categories needed to be assessed ([A + B] and [C + D]), simplifying the counting process. For this reason, we applied a grid to the two images representing the two fractions, dividing them into 48 and 63 squares, respectively. We marked and counted the specimens within a selection of squares (6 and 7, respectively) and then calculated the average number of specimens and intruders per square. These values were multiplied by the total number of squares to estimate the final counts (Figures S2 and S3).

#### 2.3.2 Damage Test

Damage assessment consisted of checking the morphological integrity of randomly selected specimens from different orders and size classes before and after sieving. To do this, we subsampled bulk *sample 1* by pouring it into a flat tray, taking an overview picture and applying a grid to divide the sample into six squares (Figure S4). From each square, we extracted 8–16 specimens from each of the four size classes (i.e. A, B, C and D). Each specimen was carefully transferred into a Petri dish using a plastic pipette to avoid any damage or soft entomological tweezers for larger specimens. We identified each insect to order or suborder level and measured body length using graph paper. Since many insects were brittle due to ethanol preservation, we measured their length in a lateral view without stretching bodies, accommodating the shape they would take while passing through the EntoSieve's mesh holes. Body length was measured from the front to the tip of the last tergite by excluding antennae, mouthparts or ovipositors, which would flex while passing through the mesh. We then assessed the intactness of each specimen by counting the number of missing and damaged antennae, legs and wings. Afterwards, each specimen was gently returned to the full‐size sample. At the end of *test 1*, we repeated the subsampling and recorded the ratio of broken and missing appendages for each size category in the same number of specimens.

All statistical analyses were performed in RStudio v. 4.4.1. We calculated the proportion of broken and missing antennae, legs and wings for each specimen and assigned a *damage score*, with broken appendages considered less severe than missing ones (broken appendage factor: 0.4; missing: 0.6). The rationale was that missing appendages can have a major effect on insect identification. We then averaged the scores of the three different appendages to obtain individual scores for broken and missing appendages of each specimen. Finally, we summed these two scores to obtain an overall *conservation status score* (*css*), which ranged from 0 to 43.34 and was standardized to a 0–100 scale. To analyse the data, we developed a generalized additive model (GAM) with a negative binomial distribution to test whether the *css* was influenced by the sieving (pre‐processing vs. post‐processing), taxonomic category or body size, including an interaction term between taxonomic category and body size. We used the *gam* function from the R package *mgcv* (version 1.9.1) (Wood and Wood 2015).

## 3 Results

### 3.1 Efficiency Tests

Examples of size‐sorted fractions are illustrated in Figures 2 and 3. Results are shown in Table 3 and Table S1.

**TABLE 3:** Results of sieving tests. For each test, we report the total number of specimens sieved, the number of correctly sieved specimens, the number and proportion of intruders, the sieving efficiency and the processing time, both for single fractions and the entire sample (Fraction: All). Additionally, we present the average efficiency of each test, with the values highlighted in bold.

Test	Fraction	Total specimens	Correctly sieved specimens	Intruders	Efficiency (%)	Average efficiency (%)	Processing time (min)
*Test 1*	A	5721	5467	254 (4.44%)	95.56%	/	6
*Test 1*	B	2753	2686	67 (2.43%)	97.57%	/	6
*Test 1*	C	1239	1219	20 (1.61%)	98.39%	/	6
*Test 1*	D	563	563	0 (0%)	100%	/	6
*Test 1*	All	10,276	9935	341 (3.32%)	/	**97.88%**	00:28:20^a^
*Test 2*	A	7242	5672	1570 (21.68%)	78.32%	/	20
*Test 2*	B	3853	3699	154 (4%)	96%	/	20
*Test 2*	C	1028	973	55 (5.35%)	94.65%	/	20
*Test 2*	D	359	359	0 (0%)	100%	/	20
*Test 2*	All	12,482	10,703	1779 (14.25%)	/	**92.24%**	01:04:40^a^
*Test 3*	[A + B]	6983	6776	207 (2.96%)	97.04%	/	20
*Test 3*	[C + D]	1387	1387	0 (0%)	100%	/	20
*Test 3*	All	8370	8163	207 (2.47%)	/	**98.52%**	00:27:20^a^

^a^ Time for collecting sieved specimens is included.

*Sample 1* consisted of 10,276 specimens, distributed as follows: 5721 specimens in size category A, 2753 in category B, 1239 in category C and 563 in category D. Only 4.44% of A‐category specimens ended up in the wrong fraction, resulting in an efficiency of 95.56% for this size category (Table 3 and Table S1). Intruders in size categories B and C were 2.43% and 1.61%, respectively, corresponding to an efficiency of 97.57% and 98.39% (Table 3). Category D exhibited 100% efficiency, as it was the last fraction remaining in the sieve container. The overall efficiency for *test 1* was 97.88%, achieved in 28 min and 20 s (6 min per mesh size, plus 10 min and 20 s for specimen recovery). *Sample 2*, used in *test 2*, contained 12,482 specimens. We sieved for 20 min per mesh size—except for MC, which required only 10 min—and collected the specimens in 15 min and 40 s, resulting in a sieving session of 1 h, 5 min and 40 s. Size category A had 7242 specimens, with 1570 (21.68%) ending up in other size categories, resulting in an efficiency of 78.32%. Category B had 3853 specimens, with 154 intruders (4%), yielding an efficiency of 96%. Category C had 1028, with 55 intruders (5.35%) and an efficiency of 94.65%. The average efficiency for *test 2* was 92.24%. *Sample 3*, used in *test 3*, consisted of 8370 specimens. Of these, 6776 were correctly fractionated into fraction [A + B] (97.04%), with 207 intruders from [A + B] appearing in the [C + D] fraction (2.96%) and 1387 specimens of category [C + D]. The efficiency for *test 3* was 98.52%. The overall processing time was 27 min and 20 s (20 min for sieving, plus 7 min and 20 s for specimen collection). Across the three tests, removing the large insects took an average of 6.37 s per specimen (Table S3).

### 3.2 Damage Test

In *test 1*, we observed comparable conservation status before and after the treatment among insects of different taxonomic groups and size categories (Table S2). The GAM revealed no significant effect of sieving on the *conservation status score* (ANOVA: χ^2^ = 0.027; *p*‐value = 0.869). Moreover, we found no significant association between observed damage and body size (ANOVA: χ^2^ = 1.361; *p*‐value = 0.243) or the interaction between taxa and body size (ANOVA: χ^2^ = 8.537; *p*‐value = 0.383), suggesting that differently sized specimens exhibited similar susceptibility to damage both before and after sieving. However, there was a significant effect of taxa on the observed damage (ANOVA: χ^2^ = 18.394; *p*‐value = 0.019), suggesting that the recorded damage likely depends on the intrinsic morphological characteristics of insect taxa.

## 4 Discussion

### 4.1 Efficiency

The EntoSieve can effectively disentangle bulk Malaise trap samples into four size fractions within 18–60 min, depending on sample heterogeneity. Given the affordability of the machine setup, multiple EntoSieves could operate in parallel. For example, fractionating five Malaise trap samples to obtain four size fractions from each would only take approximately 1 h and 30 min, including setup and retrieval time. *Test 3* simulated a shorter sieving pipeline to obtain two size fractions in a single step. In 20 min, we sorted 97.04% of small specimens (83.43% of the total) into a small fraction. We firmly believe that this reduction in time can significantly speed up single‐specimen barcoding and metabarcoding of bulk samples in large projects by replacing hours of manual labour with a single automated process.

Size‐sorting by hand and counting of individuals are time‐consuming activities, and, therefore, little is known about the size‐abundance distribution of Malaise trap samples. In this study, we obtained data on the biomass distribution of three Malaise trap samples collected over 15 days during summer in two different environments in Italy: a coastal lowland forest (*sample 1*) and a mountain grassland primarily surrounded by beech forest (*samples 2* and *3*). We found that s*amples 1* and *2* exhibited comparable ratios of size classes: 56%–58% of specimens measured less than 1.5 mm, 27%–31% measured between 1.5 and 3.0 mm, 8%–12% measured between 3.0 and 5.0 mm and 2.9%–5.5% were longer than 5.0 mm. Fractions B (1.5–3.0 mm), C (3.0–5.0 mm) and D (> 5.0 mm) achieved high efficiency levels, with 95%–100% of specimens correctly sorted (Table S1). Fraction A in *test 1* also achieved high efficiency (96%) despite its high proportion in the sample (56%). Therefore, the EntoSieve can handle large samples with over 10,000 insects, even with an uneven distribution of size classes.

In *test* 2, most intruders from the smallest size category that ended up in the second‐smallest fraction were leafhoppers (Hemiptera, Cicadellidae) (see Figure 2B). Increasing the sieving time in *test 2* did not improve the results, suggesting that the efficiency is more dependent on the sample composition than on the sieving time. The likelihood of single specimens passing through a mesh hole depends not only on their body shape and length but also on their body weight, texture (i.e. level of sclerotization) and position of appendages (i.e. antennae, legs, wings, mouthparts and ovopositor). Taxa with a compact body shape and minimal protruding appendages are less likely to become entangled and more likely to pass through the mesh. However, we observed that leafhoppers, which were extremely abundant in *sample 2*, along with a few other groups, tend to float on the water surface, possibly due to both their specific weight and hydrophobic cuticle. Aphids (Hemiptera, Sternorrhyncha) may also interfere with the sieving process because honeydew secretions appear to make them water‐repellent, causing them to form a film on the water surface. Darwin wasps (Hymenoptera, Ichneumonidae), which are particularly common in Malaise trap samples (Srivathsan et al. 2023), have sclerotized, slender and elongated bodies. They often keep their wings open and raised when preserved in ethanol and are more likely to pass through the mesh holes based on their body height rather than their length. Thus, multiple intrinsic characteristics determine the movement and orientation of specimens in the liquid, which in turn affect their likelihood of entering mesh holes.

We observed variability in sorting efficiency for *sample 1* and *sample 2*. We hypothesize that sample composition (i.e. taxonomic diversity) influences the dynamics of specimens in water. While some studies (e.g. Srivathsan et al. 2023) suggest that certain taxonomic groups consistently dominate Malaise trap samples on a global scale, significant variation may occur at smaller spatial scales. The taxonomic composition of samples collected using passive trapping methods is influenced by several factors, including seasonality (Matthews and Matthews 1970) and weather conditions (Butler et al. 1999). Additionally, variables such as trap design (Darling and Packer 1988; Uhler et al. 2022), trap placement (Darling and Packer 1988) and light exposure (Irvine and Woods 2007) can impact sample composition. To address potential challenges arising from sample composition heterogeneity, the EntoSieve is designed to be customisable with meshes varying in hole diameters. Furthermore, sieving time can be adjusted according to sample properties, stopping when most small specimens have been removed. We recommend sieving for 15–20 min per mesh size to achieve good results and recommend repeat sieving on fractions that may still contain missorted specimens, especially in the case of samples with very imbalanced body size distributions. The sieving stroke can be modulated based on taxonomic composition (e.g. higher abundances of less sclerotized, smaller and more delicate arthropods). Furthermore, extremely abundant samples can be partitioned and processed in several batches. EntoSieve can support molecular analyses of samples with varying taxonomical composition through modifications of the sieving protocol.

### 4.2 Damage Test

*Test 1* revealed that a sieving session of 18 min is not likely to damage insects, as the assessed damage before and after sieving was comparable. Furthermore, there were no significant differences in damage levels among different size categories. *Sample 1* was stored for 2 years in 96% ethanol in a dark, cool room, as no molecular analysis was planned. The breakage observed before sieving may be attributed to the effect of the highly concentrated ethanol. While higher ethanol concentrations improve DNA preservation, they can also make specimens more brittle and complicate morphological study (Marquina et al. 2021). Another potential source of breakage could be sample transportation from the field to the laboratory. To reduce specimen damage, particularly in very abundant samples, it is advisable to avoid air bubbles in the transport containers by completely filling them. Air bubbles that move during transport increase entanglement and friction, leading to more severe damage, especially on less sclerotized specimens that are more prone to breakage (Table S2). Overall, greater attention should be given to sample transport, handling and storage to avoid damage to certain taxa. Such damage can bias diversity estimates for different size classes, as loose appendages from large specimens sieved into smaller fractions can potentially lead to misleading signals.

### 4.3 Technical Advice

We recommend using the EntoSieve for sample processing because it is automated, allowing for the efficient sieving of rich bulk samples with a standardized protocol. Collecting the fractions into tubes is quick, and only a few steps in the pipeline require handling individual specimens. One manual step is the removal of extra‐large specimens (e.g. > 30 mm long), which can significantly enhance the system's efficiency by preventing large specimens from obstructing the mesh holes and disrupting the flow of smaller specimens. Large‐bodied specimens tend to entangle smaller ones, potentially skewing estimates of the large fraction due to false positives. In our samples, extra‐large specimens represented 57 individuals (0.6% of the total) in *sample 1*, 40 individuals (0.3%) in *sample 2* and 42 individuals (0.5%) in *sample 3* (Table S3). This step likely takes only a few minutes per sample but could substantially improve sequence recovery.

During sieving with the EntoSieve, specimens remain immersed in water for no longer than 60 min (i.e. 10–20 min per mesh size strikes a good balance between time and efficiency). It is unlikely that DNA quality would be compromised within this timeframe. For instance, DNA barcoding is routinely conducted on samples from pan traps, which remain submerged in water for several hours or days (deWaard et al. 2019; Creedy et al. 2020). We thus recommend using water instead of ethanol, as the system requires several litres of liquid. The use of water reduces fire hazards while significantly lowering costs, because the sorting liquid should be discarded after each use to avoid cross‐contamination from, for example, very small insects and/or bacteria. If recovering these organisms is of interest, the sieving liquid can be filtered (e.g. 20 μm cell strainer or filter paper). Using water, the EntoSieve can also be employed in field laboratories, enabling size‐sorting before shipping, which would allow for smaller containers and prevent damage in half‐empty jars.

We believe that using three mesh sizes to obtain four fractions from a bulk sample provides a favourable balance between sieving accuracy and processing time. However, depending on research requirements, fewer fractions could be generated. In metabarcoding protocols, where cost and time constraints are critical, sieving into just two fractions offers a good trade‐off between laboratory workload and taxon recovery (Elbrecht et al. 2021; Zizka et al. 2022). This approach reduces sieving time and only slightly increases sample preparation costs, which are offset by the reduced sequencing depth required to retrieve small and rare species (Elbrecht et al. 2017; Creedy et al. 2019). Our results from *test 3* simulate this workflow (Figure 3). By adjusting the protocol—specifically the mesh sizes, the number of meshes used and overall time—the EntoSieve is well suited for large‐scale projects.

Compared to the system developed by Buffington and Gates (2008), the EntoSieve allows for sorting a bulk sample into multiple size fractions, with the number of fractions adjustable based on research needs. Insects remain submerged in water for shorter periods and can be recovered midway through the sieving process, reducing prolonged submersion. Specimen retrieval is further streamlined by a semi‐automated collection step. Additionally, a coffee filter with ultra‐small holes ensures the recovery of even the smallest specimens. Elbrecht et al. (2021) tested a system designed for use with ethanol or dry specimens. In contrast, the EntoSieve employs water and minimizes specimen damage, reduces fire risk and enables efficient and gentle sieving without compromising insect morphology.

## 5 Conclusions

Malaise traps can collect extremely complex specimen samples in terms of overall abundance, species richness, taxonomic disparity and body size classes (Drake et al. 2007; Morinière et al. 2016). Size‐sorting samples is a crucial initial step in processing such bulk samples and can help with metabarcoding, as it lowers the number of false negatives for small and rare taxa (Elbrecht et al. 2017; Liu et al. 2020). Standardized methods for sieving bulk samples gently into several fractions are nevertheless still lacking (but see Buffington and Gates 2008). Here, we demonstrate that size sieving can be quick and gentle when using an up‐and‐down motion made efficient through the use of an electric motor. We thus advocate the use of sieving because it is likely to improve data quality. Avoiding biases is important and can be addressed at various stages: before wet‐lab work (e.g. size sieving), during wet‐lab work (e.g. pooling strategies) and after wet‐lab work (e.g. bioinformatics pipelines). EntoSieve will also be an essential tool for megabarcoding and mild‐lysis metabarcoding because it ensures higher voucher quality.

## Author Contributions

C.P., P.C., R.M.: conceptualization. A.A., L.W.: conception of preliminary and latest versions of the machine. L.W., N.K.: realization of the machines and the components. A.A., L.W., V.F., N.K.: testing. A.A.: statistical analysis. A.A., L.W., V.F., R.M.: visualization. A.A., L.W.: writing of original draft. C.P., P.C., R.M.: writing review and editing. C.P., P.C., R.M.: supervision. C.P., P.C., R.M.: funding acquisition. All authors have reviewed and edited the manuscript and agreed to the published version of the manuscript.

## Disclosure

*Benefit‐sharing*: The benefits generated from this research arise from the availability of our newly developed tool, which is cost‐effective and accessible to a wide audience. By sharing the tool, data and results openly on public platforms, we aim to facilitate advancements in the field and support the broader scientific community in improving and expanding molecular methodologies. This study utilized samples collected using Malaise traps from two natural parks, with all necessary collection permits obtained. All authors have reviewed and approved the manuscript, confirming the integrity and accuracy of the data presented.

## Conflicts of Interest

The authors declare no conflicts of interest.

## Supporting information


**Figure S1.** Example of count: a subsample of size fraction B (test 2). Red dots point size A specimens, blue dots point size B specimens.
**Figure S2.** Estimation of the count of specimens of size [A + B] within fraction [A + B] (test 3). Small blue dots point size [A+B] specimens.
**Figure S3.** Estimation of the count of specimens of size [A + B] and [C + D] (fraction [C + D], test 3). Blue dots point size [A+B] specimens, magenta dots point size [C + D] specimens.
**Figure S4.** Subsampling process: application of a grid on the bulk sample to select a fixed number of specimens from each square.
**Table S1.** Results of the three sieving tests performed. For each test we report the total number of specimens of each size category, the number of correctly sieved specimens, and of intruders (total and within each size fraction).
**Table S3.** Timing of the removal of large specimens and water drainage for each size fraction collection.

**Table S2.** Specimen conservation status assessment (*test 1*).

## Data Availability

All data, including code, circuit diagrams, 3D printing files and raw data of the tests, are available on the Open Science Framework (OSF) repository at the following link: https://osf.io/6t25n/.
