# Supplementary material for: EntoSieve: Automated Size‐Sorting of Insect Bulk Samples to Aid Accurate Megabarcoding and Metabarcoding
Source: Mol Ecol Resour. 2025 Mar 11;25(6):e14097. doi: 10.1111/1755-0998.14097 (PMC12225705; doi:10.1111/1755-0998.14097)
Supplement: Supplementary file 1 — Figure S1. Example of count: a subsample of size fraction B (test 2). Red dots point size A specimens, blue dots point size B specimens. Figure S2. Estimation of the count of specimens of size [A + B] within fraction [A + B] (test 3). Small blue dots point size [A+B] specimens. Figure S3. Estimation of the count of specimens of size [A + B] and [C + D] (fraction [C + D], test 3). Blue dots point size [A+B] specimens, magenta dots point size [C + D] specimens. Figure S4. Subsampling process: application of a grid on the bulk sample to select a fixed number of specimens from each square. Table S1. Results of the three sieving tests performed. For each test we report the total number of specimens of each size category, the number of correctly sieved specimens, and of intruders (total and within each size fraction). Table S3. Timing of the removal of large specimens and water drainage for each size fraction collection. [file MEN-25-e14097-s002.docx]

**EntoSieve: automated size-sorting of insect bulk samples to aid accurate megabarcoding and metabarcoding**

Aleida Ascenzi, Lorenz Wührl, Vivian Feng, Nathalie Klug, Christian Pylatiuk, Pierfilippo Cerretti, Rudolf Meier


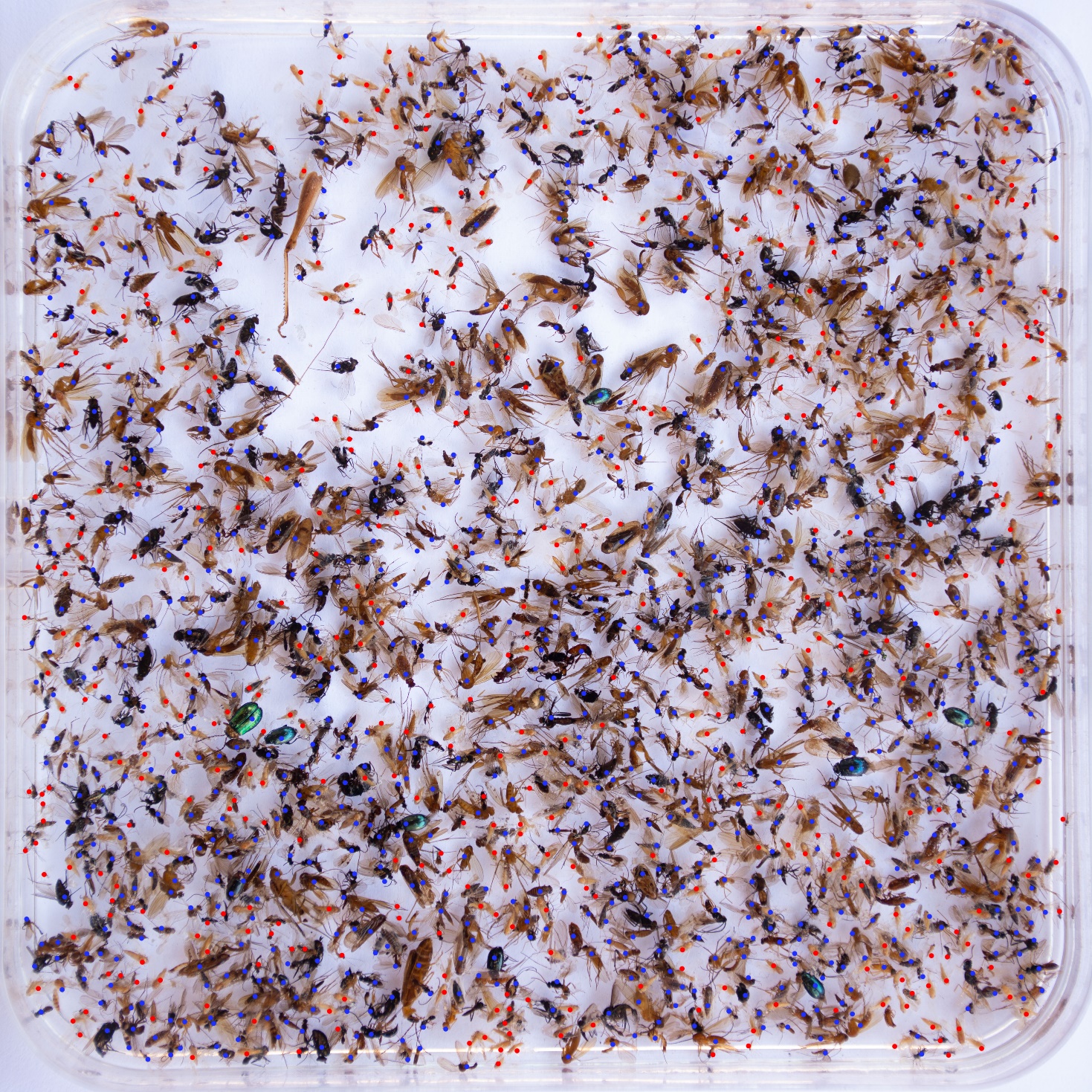


***FIGURE S1.*** *Example of count: a subsample of size fraction B (test 2). Red dots point size A specimens, blue dots point size B specimens.*


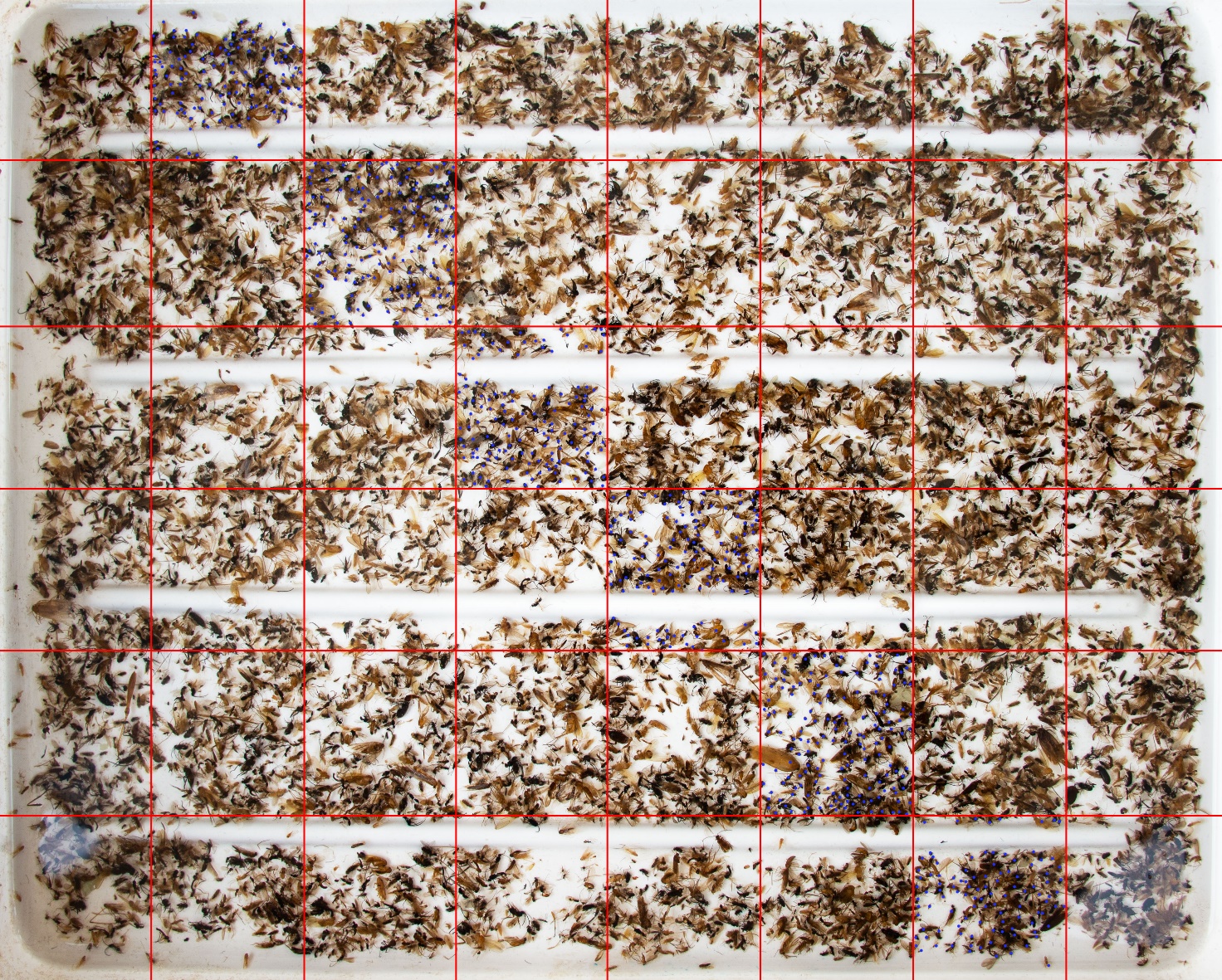


***FIGURE S2.*** *Estimation of the count of specimens of size [A+B] within fraction [A+B] (test 3). Small blue dots point size [A+B] specimens.*


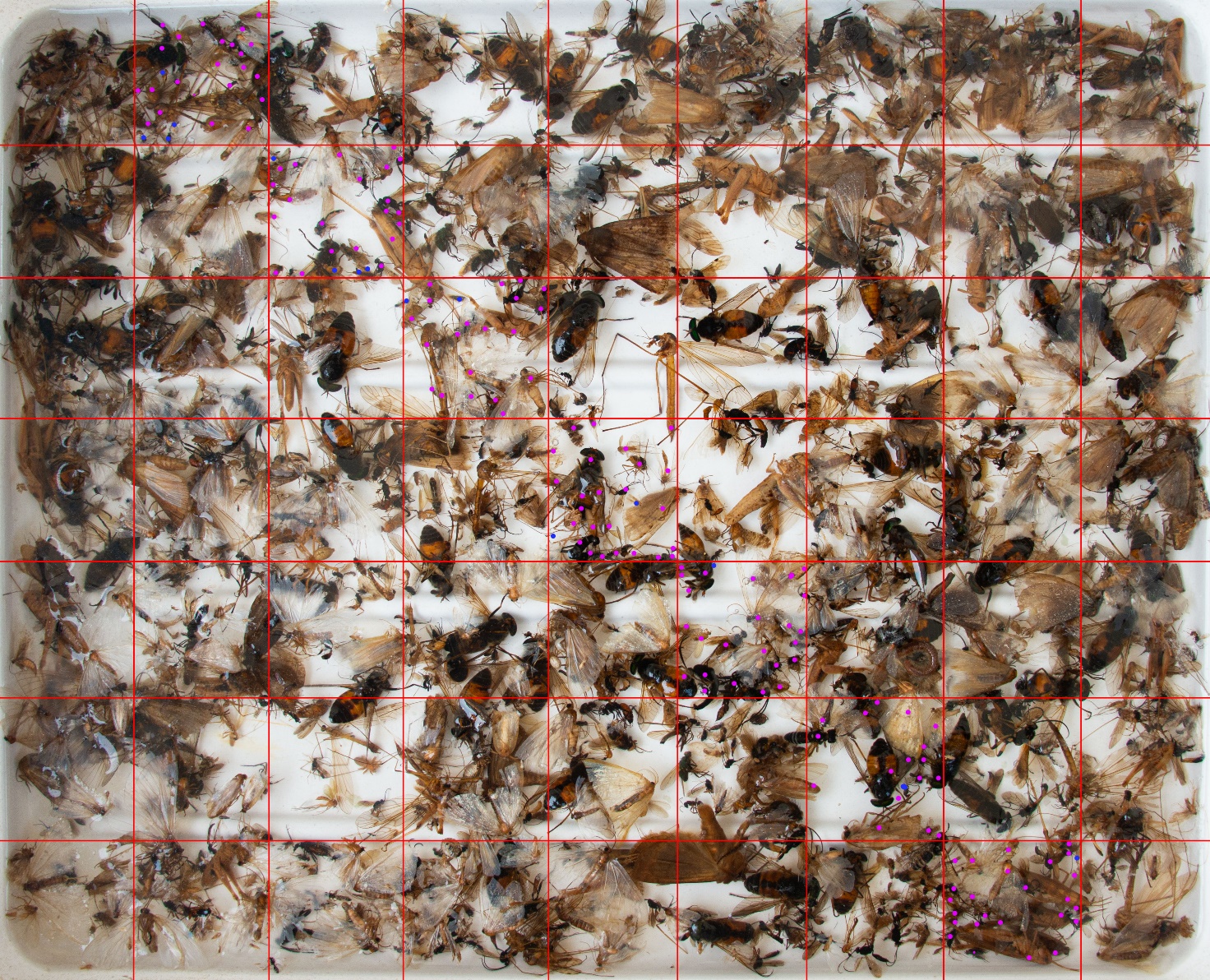


***FIGURE S3.*** *Estimation of the count of specimens of size [A+B] and [C+D] (fraction [C+D], test 3). Blue dots point size [A+B] specimens, magenta dots point size [C+D] specimens.*


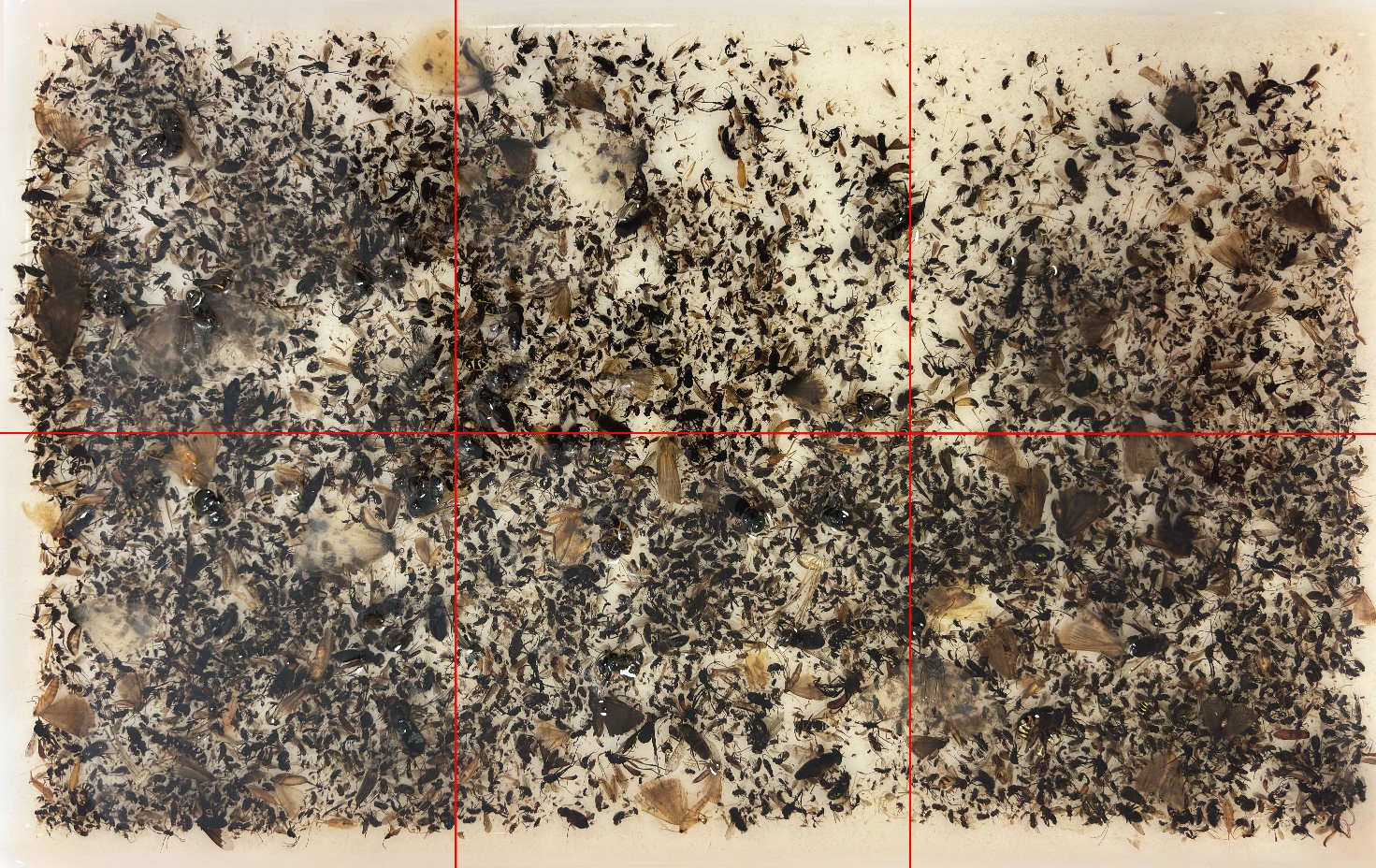


***FIGURE S4.*** *Subsampling process: application of a grid on the bulk sample to select a fixed number of specimens from each square.*

***Table S1.*** *Results of the three sieving tests performed. For each test we report the total number of specimens of each size category, the number of correctly sieved specimens, and of intruders (total and within each size fraction).*

| *Category* | *Test 1* | *Test 2* | *Test 3* |
| --- | --- | --- | --- |
| *A specimens* | *5,721* | *7,242* |  |
| *A correctly sieved* | *5,467* | *5,672* |  |
| *A intruders* | *254* | *1,570* |  |
| *A in B* | *191* | *1,486* |  |
| *A in C* | *50* | *84* |  |
| *A in D* | *13* | *0* |  |
| *B specimens* | *2,753* | *3,853* |  |
| *B correctly sieved* | *2,686* | *3,699* |  |
| *B intruders* | *67* | *154* |  |
| *B in C* | *54* | *154* |  |
| *B in D* | *13* | *0* |  |
| *[A + B] specimens* |  |  | *6,983* |
| *[A + B] correctly sieved* |  |  | *6,776* |
| *[A + B] intruders* |  |  | *207* |
| *[A + B] in D* |  |  | *207* |
| *[C + D] specimens* |  |  | *1,387* |
| *[C + D] correctly sieved* |  |  | *1,387* |
| *[C + D] intruders* |  |  | *0* |
| *C specimens* | *1239* | *1028* |  |
| *C correctly sieved* | *1219* | *973* |  |
| *C intruders* | *20* | *55* |  |
| *C in D* | *20* | *55* |  |
| *D specimens* | *563* | *359* |  |
| *D correctly sieved* | *563* | *359* |  |
| *D intruders* | *0* | *0* |  |
| *Total intruders* | *341* | *1779* |  |
| *Total correctly sieved* | *9935* | *10703* |  |
| *Total specimens* | *10276* | *12482* | *8370* |

***Table S3.*** *Timing of the removal of large specimens and water drainage for each size fraction collection.*

|  | *test 1* | *test 2* | *test 3* |
| --- | --- | --- | --- |
| *activity* | *Manual removal of large specimens* | | |
| *timing* | *00:05:25* | *00:04:42* | *00:04:38* |
| *specimens* | *55xLepidoptera, 1xLepidoptera larvae, 1xVespidae* | *40xLepidoptera* | *29xLepidoptera, 10xOrthoptera, 3xVespidae* |
|  | *Water draining (h)* | | |
| *fraction A* | *00:03:30* | *00:07:25* |  |
| *fraction B* | *00:02:40* | *00:03:45* |  |
| *fraction C* | *00:02:10* | *00:02:20* |  |
| *fraction D* | *00:02:00* | *00:02:10* |  |
| *fraction [A+B]* |  |  | *00:04:45* |
| *fraction [C+D]* |  |  | *00:02:35* |
